# Clinical practice of early screening and risk-stratified management for oral potentially malignant disorders

**DOI:** 10.3389/fonc.2025.1697676

**Published:** 2025-10-08

**Authors:** Beibei Ge, Xinqiang Zhu, Meimei Ma, Yingxin Ju, Yannan Ma, Yong Song

**Affiliations:** ^1^ Department of Stomatology, The Affiliated Suqian Hospital of Xuzhou Medical University, Suqian ,Jiangsu, China; ^2^ Department of General Surgery, The Affiliated Suqian Hospital of Xuzhou Medical University, Suqian ,Jiangsu, China; ^3^ Department of Pathology, The Affiliated Suqian Hospital of Xuzhou Medical University, Suqian ,Jiangsu, China

**Keywords:** oral potentially malignant disorders, early detection, risk stratification, malignant transformation, multidisciplinary team

## Abstract

**Objective:**

To evaluate the application value of standardized screening and risk-stratified management in the clinical practice of Oral Potentially Malignant Disorders (OPMDs) for improving early diagnosis rates and optimizing intervention strategies.

**Methods:**

A total of 312 OPMD patients diagnosed between January 2017 and December 2022 were enrolled. A screening pathway of “initial screening (questionnaire + visual/tactile examination) - refined screening (pathological biopsy)” was established. Risk stratification (low, intermediate, high) was performed using a modified Proliferative Verrucous Leukoplakia (PVL)-based scoring system. Multidisciplinary team (MDT) management was implemented. Risk factors for malignant transformation were analyzed using Cox regression.

**Results:**

The high-risk group had a significantly higher malignant transformation rate than the intermediate and low-risk groups (12.5% vs. 3.5% vs. 0%, P<0.001), with a shorter median time to transformation (23.4 months). Severe epithelial dysplasia (HR = 6.24), lesions located in the tongue ventral/floor of mouth (HR = 3.34), and betel quid chewing history (HR = 2.62) were identified as independent risk factors. The MDT model achieved a 2-year cancer-free survival rate of 91.4% in high-risk patients and improved follow-up compliance to 83.3%.

**Conclusion:**

An OPMD management model based on risk stratification and MDT collaboration can effectively identify high-risk patients, optimize intervention timing, and improve prognosis. This model is suitable for promotion in primary care hospitals.

## 1 Introduction

Head and neck squamous cell carcinoma (HNSCC) ranks as the sixth most common cancer globally, with approximately 900,000 cases annually, including over 400,000 new cases and 178,000 deaths (1). According to the International Agency for Research on Cancer (IARC), oral cavity cancer was estimated to affect about 400,000 people and cause 178,000 deaths in 2020, ranking 16th globally in both incidence and mortality (2). Oral squamous cell carcinoma (OSCC) accounts for over 90% of all oral malignancies (3), is the most common oral cancer (4), and demonstrates a higher incidence in males. Furthermore, global incidence rates show geographical variation, attributable to differing exposures to carcinogenic risk factors such as tobacco (including smoking and smokeless forms) (5). Compared to breast cancer, OSCC has a poorer prognosis, with a 5-year survival rate of around 50% and annual treatment costs reaching up to 2 billion USD (6).

Early screening and intervention for OPMDs (7) are widely recognized as the “golden window” for blocking the progression to cancer and improving outcomes (8). The clinical presentation of both OPMDs and OSCC varies geographically, even within the same country, likely due to shared habits and cultural factors like excessive use of tobacco products, leading to a higher prevalence of tobacco-related lesions. A deeper understanding of regional patterns and behaviors could aid in developing preventive and therapeutic strategies (9). However, current OPMD management faces three core challenges: 1) Weak screening awareness and lack of standardization: Primary care institutions often lack systematic screening training, resulting in high missed diagnosis rates; traditional reliance on “visual and tactile examination” is highly subjective, necessitating standardized procedures and supportive technologies. 2) Ambiguous risk stratification and delayed intervention, leading to overtreatment of low-risk patients and inadequate follow-up for high-risk patients. 3) Absence of a Multidisciplinary Team (MDT) mechanism. Addressing these challenges, international leading guidelines (e.g., NCCN, AAOMP) emphasize that building an integrated pathway of “screening - risk assessment - stratified intervention - long-term follow-up” is crucial[10-11&]. Developing an OPMD management model that is sensitive, operable, and aligns with the practical resource constraints of primary and tertiary hospitals in China holds significant practical importance.

## 2 Materials and methods

### 2.1 Study population

#### 2.1.1 Inclusion criteria

Patients clinically diagnosed with OPMD in the Department of Oral Medicine of our hospital between January 2017 and December 2022. Patients provided signed informed consent.

#### 2.1.2 Exclusion criteria

Previously diagnosed oral cancer, history of head and neck radiotherapy, inability to comply with follow-up.

Sample size: 312 patients were ultimately included.

### 2.2 Research methods

#### 2.2.1 Standardized screening pathway

##### 2.2.1.1 Initial screening (general dentist)

###### 2.2.1.1.1 Questionnaire

Record history of smoking, alcohol consumption, betel quid chewing, HPV exposure, systemic diseases, and symptom duration.

Visual & Tactile Examination: Standardized recording of lesion location, size, color, texture, borders, and bleeding tendency.

###### 2.2.1.1.2 Preliminary risk assessment

Application of a simple checklist (e.g., red/white patch >1cm + risk factors = referral to specialist).

##### 2.2.1.2 Specialist refined screening (oral medicine)

Pathological Gold Standard: Perform incisional biopsy on suspected cases (biopsy principle: most abnormal area + border zone).

#### 2.2.2 Risk assessment and stratified management

A scoring system was designed based on features associated with Proliferative Verrucous Leukoplakia (PVL) (10) (**Table 1**). Based on the total score, patients were stratified into Low-risk (0–4 points), Intermediate-risk (5–8 points), and High-risk (≥9 points) groups.

**Table 1:** Original PVL-based scoring system.

Scoring item	Scoring criteria	Points
Pathological Grade	No dysplasia	0
	Mild dysplasia	3
	Moderate/Severe dysplasia	6
Lesion Site	Lip, Gingiva, Palate, Buccal Mucosa	0
	Floor of mouth, Ventral tongue, Lateral tongue, Soft palate	5
Lesion Size	≤ 20 mm	0
	> 20 mm	4
Morphological Type	Homogeneous	0
	Non-homogeneous (Nodular, Verrucous, Erosive)	3

#### 2.2.3 Multidisciplinary team model

Collaborating Departments: Oral Pathology, Head and Neck Surgery, Oncology, Otolaryngology.

##### 2.2.3.1 Process

Initial diagnosis in Oral Medicine + Biopsy confirmation → MDT consultation for high-risk cases (determine treatment plan) → Treatment implementation (Oral Medicine/Surgery) → Oncology follow-up (if malignant transformation occurs) → Shared data platform.

#### 2.2.4 Follow-up protocol

Low-risk: 6-month review.

Intermediate/High-risk: 3-month review (clinical + photographic documentation).

Annual review: Indications for repeat biopsy (lesion change, worsening symptoms).

### 2.3 Evaluation indicators

#### 2.3.1 Primary indicators

OPMD detection rate, proportion of high-risk patients, malignant transformation rate, time to transformation.

#### 2.3.2 Secondary indicators

Specificity of adjunctive tests, patient follow-up compliance rate, MDT consultation implementation rate, treatment effective rate (lesion reduction/stabilization rate).

### 2.4 Statistical analysis

Statistical analysis was performed using SPSS software (version 23.0). Measurement data were analyzed using independent samples t-test. Count data were analyzed using the Chi-square test (χ² test) to examine the influence of various factors on outcomes (significance level α=0.05). Binary logistic regression was used to assess the cross-sectional associations between patient characteristics and the presence of high-grade dysplasia Factors found to be statistically significant were included as independent variables in a Logistic regression analysis (significance level α=0.05). univariable and multivariable Cox proportional hazards regression models were employed to calculate hazard ratios (HRs) and 95% confidence intervals.

As this was a retrospective study, a formal sample size calculation was not performed *a priori*. Instead, we aimed to include all eligible cases within the specified timeframe to maximize the sample size and statistical power.”

### 2.5 Ethics and consent

We conducted the study in accordance with the Declaration of Helsinki, and the patients provided signed informed consent. The research protocol was approved by the Institutional Review Committee and Human Ethics Committee of Suqian Hospital affiliated with Xuzhou Medical University.

## 3 Results

### 3.1 Baseline characteristics of the study population

**Figure 1:**
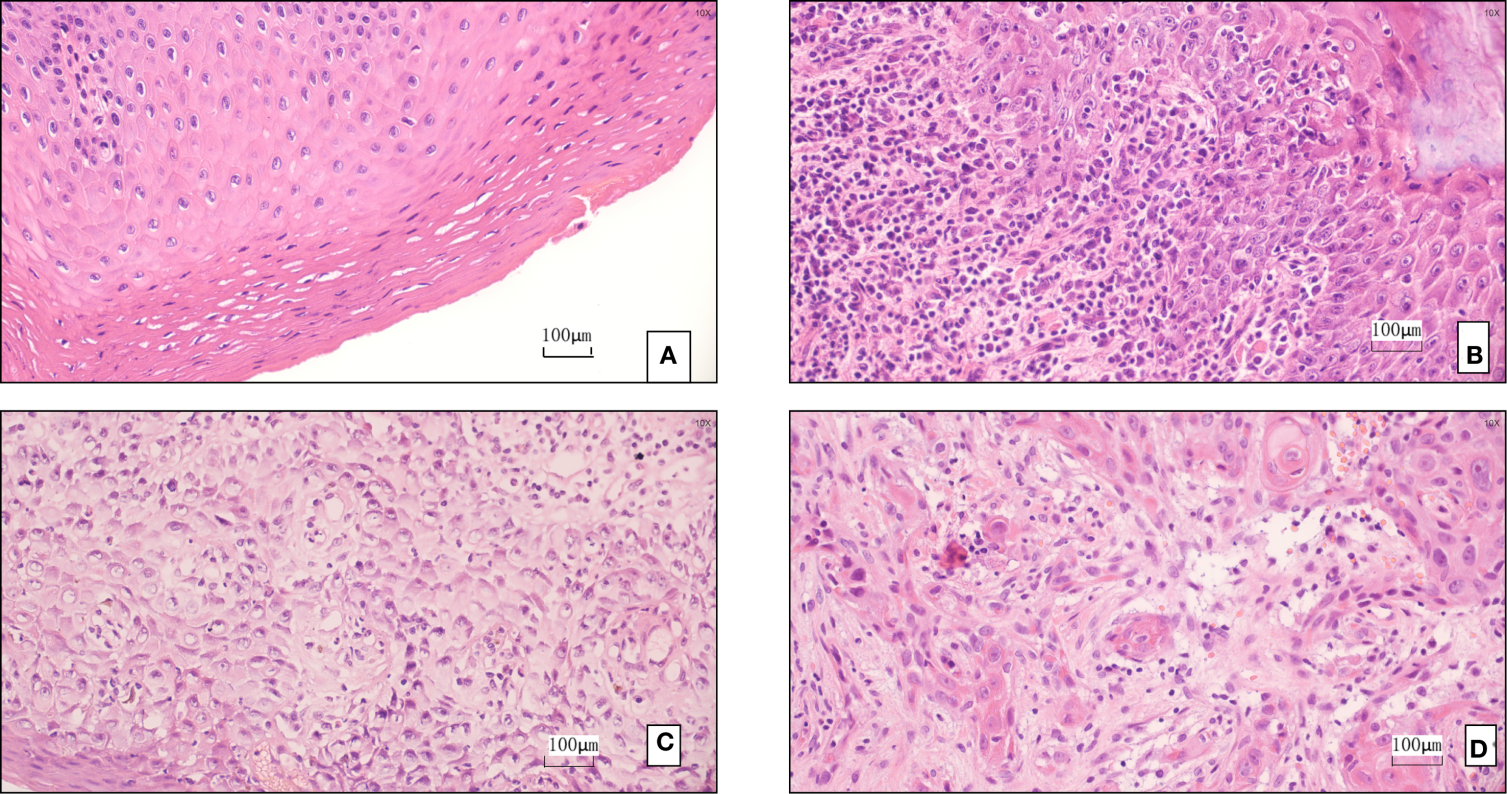
**(A)** Pathological finding: leukoplakia of the mucosa. **(B)** The pathology suggests oral lichen planus. **(C)** The pathology indicates moderate to severe dysplasia. **(D)** The pathology indicates squamous cell carcinoma.

This study included 312 OPMD patients diagnosed between January 2017 and December 2022, with a mean follow-up time of 28.6 ± 9.3 months (range: 12–48 months). Patients were predominantly male (65.4%) and middle-aged or elderly (50–69 years, 58.0%). The main OPMD types were oral leukoplakia (52.9%) and Lichen planus (10.3%) (**Figures 1A, B**). 6.7% had a history of betel quid chewing (reflecting significant regional characteristics). Pathological biopsy revealed moderate to severe epithelial dysplasia in 23.1% of cases (**Table 2**) (**Figure 1C**).

**Table 2:** Baseline characteristics of OPMD patients (n=312).

Characteristic	Category	Number	Percentage (%)
Gender	Male	204	65.4
	Female	108	34.6
Age (years)	<40	68	21.8
	40-49	82	26.3
	50-69	181	58.0
	≥70	21	6.7
OPMD Type	Leukoplakia	165	52.9
	Submucous Fibrosis	102	32.7
	Erosive Lichen Planus	32	10.3
	Erythroplakia	13	4.2
Lesion Site	Buccal Mucosa	158	50.6
	Tongue Ventral/FOM*	89	28.5
	Gingiva	65	20.8
Pathological Grade	No dysplasia	128	41.0
	Mild dysplasia	112	35.9
	Moderate dysplasia	52	16.7
	Severe dysplasia	20	6.4
Main Risk Factors	Smoking (>10 pack-years)	187	59.9
	Alcohol (>40g/day)	121	38.8
	Betel Quid Chewing	21	6.7

*FOM, Floor of mouth.

### 3.2 Risk stratification and malignant transformation outcomes

Stratification based on the modified PVL model resulted in: Low-risk group (n=102, 32.7%), Intermediate-risk group (n=141, 45.2%), and High-risk group (n=69, 22.1%). During follow-up, 21 cases underwent malignant transformation (overall transformation rate 6.7%) (**Figures 1D**). The transformation rate was significantly higher in the High-risk group (12.5% vs. 3.5% in Intermediate vs. 0% in Low-risk, P<0.001). The median time to transformation was significantly shorter in the High-risk group (23.4 months, 95% CI: 18.6-28.2) compared to the Intermediate-risk group (Log-rank χ²=18.7, P<0.001) (**Table 3**). Lesions located in the tongue ventral/floor of mouth had the highest transformation rate (11.2%), followed by those with severe dysplasia (10.0%) (**Table 4**). Notably, a specific high-risk subgroup was identified: patients with a history of betel quid chewing, lesions located in the tongue ventral/floor of mouth, and a pathological diagnosis of mild dysplasia (n=22). Despite the low pathological grade, their cumulative transformation rate was 9.1% (2/22). Based on this finding, all such patients were included in the high-risk management category.

**Table 3:** Malignant transformation by risk stratification.

Risk stratification	n	Transformation n	Rate (%)	Median time to transformation (months)
Low-risk	102	0	0.0	–
Intermediate-risk	141	5	3.5	34.2 (28.5-39.8)
High-risk	69	16	12.5*	23.4 (18.6-28.2)*
Total	312	21	6.7	26.8 (22.1-31.5)

*High-risk vs. Intermediate risk: χ²=9.32, P=0.002; Time comparison: Log-rank χ²=18.7, P<0.001.

**Table 4:** Transformation by clinicopathological features.

Feature	Total n	Transformation n	Cumulative rate (%)	Mean FU (mo)
Lesion Site				
Tongue Ventral/FOM	89	10	11.2	28.5
Buccal Mucosa	158	8	5.1	29.1
Gingiva	65	3	4.6	27.8
Pathological Grade				
Severe dysplasia	20	2	10.0	26.2
Moderate dysplasia	52	3	5.8	28.0
Mild/No dysplasia	240	16	6.7	29.0

FU, Follow-up.

### 3.3 Analysis of risk factors for malignant transformation

Multivariable Cox proportional hazards regression analysis identified the following independent risk factors for transformation: severe epithelial dysplasia (strongest predictor, HR = 6.24, 95% CI: 2.73-14.25, P<0.001); lesions located in the tongue ventral/floor of mouth (HR = 3.34, 95% CI: 1.55-7.19, P = 0.002); history of betel quid chewing (HR = 2.62, 95% CI: 1.25-5.47, P = 0.010). The model passed the Schoenfeld residual test (P = 0.32), indicating proportionality of hazards. The -2 Log Likelihood was 142.6 (**Table 5**).

**Table 5:** Cox regression analysis of factors influencing OPMD transformation (n=312).

Variable	β value	HR (95% CI)	P value
Severe Dysplasia	1.832	6.24 (2.73-14.25)	<0.001**
Tongue Ventral/FOM	1.205	3.34 (1.55-7.19)	0.002**
Betel Quid Chewing	0.963	2.62 (1.25-5.47)	0.010*
Age ≥60 years	0.541	1.72 (0.98-3.02)	0.060
Smoking (>10 pack-yr)	0.317	1.37 (0.82-2.31)	0.232

*P<0.05, **P<0.01.

### 3.4 Effectiveness of multidisciplinary team management

The MDT implementation rate in the high-risk group was 89.9% (62/69). MDT significantly improved patient compliance (follow-up attendance rate: MDT group 83.3% vs. non-MDT group 52.9%, P = 0.008). High-risk patients who underwent surgical resection achieved a 2-year cancer-free survival rate of 91.4%, compared to 68.2% in those who did not undergo surgery (P = 0.003). The treatment plan was modified following MDT consultation in 27.4% of cases (17/62), primarily involving extended excision margins or combined immune preventive strategies.

## 4 Discussion

### 4.1 Value of standardized screening in primary care practice: optimizing low-cost technology combinations

Oral cancer (OC) is an uncommon malignancy in Western countries but ranks among the most common cancers in some high-risk regions globally. It is largely preventable, as major risk factors like smoking, alcohol, and betel quid chewing are modifiable behaviors. Given its high mortality, early diagnosis is crucial (11). This study confirms that a standardized screening pathway based on structured visual/tactile examination and targeted adjunctive tests is vital in primary care settings. Tolonium chloride (Toluidine Blue) staining, as reported elsewhere, demonstrates high specificity for dysplasia, making it a key tool for precisely guiding biopsy location and avoiding missed diagnoses from random sampling (12). Liquid-based cytology can significantly reduce unnecessary biopsies in low-risk patients (13). These findings address a current controversy in OPMD screening: in resource-limited areas, optimizing combinations of existing tools is more feasible than pursuing advanced technologies. We recommend “staining + cytology” as a fundamental screening package suitable for general practitioners due to its simplicity. Although evidence is lacking to support adjunctive techniques like Tolonium chloride staining or fluorescence imaging as screening tools for reducing oral cancer mortality (14), they represent low-cost options suitable for widespread use. Certainly, the combined use of autofluorescence, chemiluminescence, and Tolonium chloride (TBlue) may be helpful in specialist clinics for assessing lesions, though their accuracy compared to conventional oral examination and biopsy requires further discussion; more research is needed to examine their role in primary care screening (15). Our model is cost-effective yet skill-oriented, ensuring precise operations that are easy to promote.

### 4.2 Clinical significance of the risk stratification model: from “empirical judgment” to “quantified decision-making”

This study innovatively integrated the PVL-based index with regional high-risk factors (tongue ventral lesions, betel quid history) to construct a stratification model (16), and validated its predictive efficacy in a large sample: the high-risk group had a 12.5-fold higher transformation risk than the low-risk group (**Table 3**), with significantly earlier transformation (median 23.4 vs. 34.2 months, P<0.001). The Cox model further identified severe dysplasia (HR = 6.24), tongue ventral lesions (HR = 3.34), and betel quid history (HR = 2.62) as independent predictors (**Table 5**). The value of this model lies in: 1) *Resolving clinical decision-making dilemmas:* The traditional management model relying solely on pathological grade overlooks the synergistic carcinogenic effects of behavioral (betel quid) and anatomical (tongue ventral) factors. 2) *Enabling precise resource allocation:* Matching follow-up intensity to risk level (high-risk: 3 months vs. low-risk: 6 months) avoids waste of medical resources. 3) *Advancing prevention:* Patients with the combination of “betel quid + tongue ventral leukoplakia” were managed as high-risk even with mild dysplasia (transformation rate 9.1% in this subgroup). This underscores that oral carcinogenesis is a multifactorial process; the biological toxicity of betel quid combined with the anatomical vulnerability of the tongue ventral site constitutes an independent risk driver not always captured by current pathological grading systems. Therefore, a decision-making model integrating clinical, behavioral, and anatomical features is a crucial supplement to traditional pathological grading, facilitating earlier and more precise intervention. Future efforts should focus on developing digital risk calculators integrated into electronic medical record systems for automatic alerts. Currently, there is no conclusive evidence for effective treatments preventing oral cancer development in OPMDs like leukoplakia; larger, longer-term trials are needed to fully assess the impact of OPMD treatment on oral cancer risk (17).

### 4.3 Core role of the multidisciplinary team system: an essential strategy to break down specialty barriers

In this study, MDT implementation improved compliance in high-risk patients by 30.4% and achieved a 2-year cancer-free survival rate of 91.4% post-surgical intervention, highlighting its indispensable value. In 82% of the key cases where the treatment plan was revised (27.4% revision rate), the revision addressed underestimation of lesion aggressiveness by Oral Medicine (e.g. erythroplakia with micro-invasion requiring wider excision). Involvement of Oncology led to chemoprevention strategies (e.g. topical retinoids) that reduced transformation risk in non-surgical high-risk patients. These findings address the fundamental contradiction in OPMD management: Oral Medicine excels at early diagnosis but lacks definitive treatment means, while Surgery/Oncology specializes in radical treatment but often encounters late-stage cases. Our MDT model addresses this through three core designs: 1) *Standardized referral trigger mechanism:* Automatic MDT consultation triggered by severe dysplasia/PVL high-risk score. 2) *Shared electronic decision board:* Integrates clinical, imaging, and pathological data for real-time assessment. 3) *Patient navigation throughout the process:* Dedicated nurses coordinate referrals. This model provides a replicable management framework for regional OPMD prevention and treatment networks.

## 5 Conclusion

This study demonstrates the clinical feasibility of early OPMD control through a three-tiered system: “Accessible Screening → Data-Driven Stratification → Integrated Intervention.” Future directions should focus on: ① Exploring AI-assisted lesion identification; ② Validating the predictive value of molecular markers; ③ Promoting the inclusion of OPMD management into national chronic disease prevention and control systems. The generalizability of our findings may be limited by the single-center, retrospective nature of the study and the sample size, warranting future validation in multi-center prospective studies.

## Data Availability

The datasets presented in this study can be found in online repositories. The names of the repository/repositories and accession number(s) can be found in the article/supplementary material.

## References

[1] Ferlay J Colombet M Soerjomataram I Mathers C Parkin DM Piñeros M. Estimating the global cancer incidence and mortality in 2018: GLOBOCAN sources and methods. Int J Cancer. (2019) 144:1941–53. doi: 10.1002/ijc.31937, PMID: 30350310

[2] Sung H Ferlay J Siegel RL Laversanne M Soerjomataram I Jemal A. Global cancer statistics 2020: GLOBOCAN estimates of incidence and mortality worldwide for 36 cancers in 185 countries. CA Cancer J Clin. (2021) 71:209–49. doi: 10.3322/caac.21660, PMID: 33538338

[3] Bagan J Sarrion G Jimenez Y. Oral cancer: clinical features. Oral Oncol. (2010) 46:414–7. doi: 10.1016/j.oraloncology.2010.03.009, PMID: 20400366

[4] Chamoli A Gosavi AS Shirwadkar UP Wangdale KV Behera SK Kurrey NK. Overview of oral cavity squamous cell carcinoma: Risk factors, mechanisms, and diagnostics. Oral Oncol. (2021) 121:105451. doi: 10.1016/j.oraloncology.2021.105451, PMID: 34329869

[5] Mroueh R Nevala A Haapaniemi A Pitkäniemi J Salo T Mäkitie AA. Risk of second primary cancer in oral squamous cell carcinoma. Head Neck. (2020) 42:1848–58. doi: 10.1002/hed.26107, PMID: 32057158

[6] D’Silva NJ Ward BB. Tissue biomarkers for diagnosis & management of oral squamous cell carcinoma. Alpha Omegan. (2007) 100:182–9. doi: 10.1016/j.aodf.2007.10.014, PMID: 18335816

[7] Pritzker KPH Darling MR Hwang JT Mock D. Oral Potentially Malignant Disorders (OPMD): What is the clinical utility of dysplasia grade? Expert Rev Mol Diagn. (2021) 21:289–98. doi: 10.1080/14737159.2021.1898949, PMID: 33682567

[8] Warnakulasuriya S. Clinical features and presentation of oral potentially Malignant disorders. Oral Surg Oral Med Oral Pathol Oral Radiol. (2018) 125:582–90. doi: 10.1016/j.oooo.2018.03.011, PMID: 29673799

[9] Kumar GK Abidullah M Elbadawi L Dakhil S Mawardi H. Epidemiological profile and clinical characteristics of oral potentially Malignant disorders and oral squamous cell carcinoma: A pilot study in Bidar and Gulbarga Districts, Karnataka, India. J Oral Maxillofac Pathol. (2019) 23:90–6. doi: 10.4103/jomfp.JOMFP_116_18, PMID: 31110423 PMC6503790

[10] Thompson LDR Fitzpatrick SG Müller S Eisenberg E Upadhyaya JD Lingen MW. Proliferative verrucous leukoplakia: an expert consensus guideline for standardized assessment and reporting. Head Neck Pathol. (2021) 15:572–87. doi: 10.1007/s12105-020-01262-9, PMID: 33415517 PMC8134585

[11] Abati S Bramati C Bondi S Lissoni A Trimarchi M. Oral cancer and precancer: A narrative review on the relevance of early diagnosis. Int J Environ Res Public Health. (2020) 17:9160. doi: 10.3390/ijerph17249160, PMID: 33302498 PMC7764090

[12] Vijayakumar V Reghunathan D Edacheriyan B Mukundan A. Role of toluidine blue staining in suspicious lesions of oral cavity and oropharynx. Indian J Otolaryngol Head Neck Surg. (2019) 71:142–6. doi: 10.1007/s12070-017-1161-y, PMID: 31741949 PMC6848367

[13] Hegde U Sheshanna SH Jaishankar HP Shashidara R. Liquid-based cytology in the diagnosis of oral pre-cancer, cancer, and other oral lesions - A narrative review. J Oral Maxillofac Pathol. (2024) 28:535–43. doi: 10.4103/jomfp.jomfp_332_24, PMID: 39949686 PMC11819620

[14] Brocklehurst P Kujan O O’Malley LA Ogden G Shepherd S Glenny AM. Screening programmes for the early detection and prevention of oral cancer. Cochrane Database Syst Rev. (2013) 2013:Cd004150. doi: 10.1002/14651858.CD004150.pub4, PMID: 24254989 PMC8078625

[15] Awan KH Morgan PR Warnakulasuriya S. Assessing the accuracy of autofluorescence, chemiluminescence and toluidine blue as diagnostic tools for oral potentially Malignant disorders–a clinicopathological evaluation. Clin Oral Investig. (2015) 19:2267–72. doi: 10.1007/s00784-015-1457-9, PMID: 25804887

[16] Warnakulasuriya S Chen THH. Areca nut and oral cancer: evidence from studies conducted in humans. J Dent Res. (2022) 101:1139–46. doi: 10.1177/00220345221092751, PMID: 35459408 PMC9397398

[17] Lodi G Franchini R Warnakulasuriya S Varoni EM Sardella A Kerr AR. Interventions for treating oral leukoplakia to prevent oral cancer. Cochrane Database Syst Rev. (2016) 7:Cd001829. doi: 10.1002/14651858.CD001829.pub4, PMID: 27471845 PMC6457856

